# Synthesis of MnSe-Based GO Composites as Effective Photocatalyst for Environmental Remediations

**DOI:** 10.3390/nano13040667

**Published:** 2023-02-08

**Authors:** Violeta Jevtovic, Afaq Ullah Khan, Zainab M. Almarhoon, Kamran Tahir, Salman Latif, Fahad Abdulaziz, Karma Albalawi, Magdi E. A. Zaki, Violeta Rakic

**Affiliations:** 1 Department of Chemistry, College of Science, University of Ha’il, Ha’il 81451, Saudi Arabia; 2 State Key Laboratory of Chemical Resource Engineering, School of Science, Beijing University of Chemical Technology, Beijing 100029, China; 3 School of Chemistry and Chemical Engineering, Jiangsu University, 301 Xuefu Road, Zhenjiang 212013, China; 4 Chemistry Department, College of Science, King Saud University, P.O. Box 2455, Riyadh 11451, Saudi Arabia; 5 Institute of Chemical Sciences, Gomal University, Dera Ismail Khan 29050, Pakistan; 6 Department of Chemistry, Faculty of Science, University of Tabuk, Tabuk 71491, Saudi Arabia; 7 Department of Chemistry, Faculty of Science, Imam Mohammad Ibn Saud Islamic University, Riyadh 11623, Saudi Arabia; 8 Department of Agriculture and Food Technology Prokuplje, Academy of Vocational Studies of South Serbia, 18400 Prokuplje, Serbia

**Keywords:** manganese selenide, GO, methylene blue, organic dye, catalytic activity

## Abstract

In this work, a manganese selenide/graphene oxide (MnSe/GO)-based composite was prepared for wet-chemical assisted method against organic dye; herein, methylene blue (MB) dye removal from the water was employed as a metal selenide-based photocatalyst. The synthesized MnSe/GO composite was systematically characterized by X-ray diffraction (XRD), Fourier transform electron microscopy (FTIR), scanning electron microscopy (SEM), energy dispersive X-ray spectroscopy (EDS), and UV-visible diffuse reflectance spectroscopy (UV-vis. DRS). The structural characteristic revealed the adequate synthesis of the sample with good crystallinity and purity of the obtained products. The morphological analysis indicates the formation of MnSe nanoflakes composed of tiny particles on their surface. At the same time, the GO nanosheets with high aggregation were formed, which may be due to the van der Waals forces. The bond interaction and compositional analysis studies confirmed and supported the structural findings with high purity. The optical analysis showed the bandgap energies of MnSe and their composites MnSe (1.7 eV), 7% GO-MnSe (2.42 eV), 14% GO-MnSe (2.6 eV), 21% GO-MnSe (3.02 eV), and 28% GO-MnSe (3.24 eV) respectively, which increase the bandgap energy after GO and MnSe recombination. Among different contents, the optimized 21% GO-MnSe composite displayed enhanced photocatalytic properties. For instance, a short time of 90 min was taken compared with other concentrations due to the narrow bandgap of MnSe and the highly conductive charge carrier’s support, making the process to remove MB from water faster. These results show that the selenide-based photocatalyst can be an attractive candidate for future advanced photocatalysis applications.

## 1. Introduction

Due to their distinctive characteristics and possible uses in various disciplines, one-dimensional (1D) semiconductor nanomaterials such as nanorods, nanobelts, and nanowires have attracted growing attention [1,2]. One-dimensional (1D) nanostructured components self-assemble three-dimensional (3D) architectures that exhibit properties of 1D nanomaterials, including the quantum confinement, little impact, and surface effect [3], and also some innovative products that are a result of 3D assemblies, such as the quantum coupling effect and synergistic effect [4]. To achieve their novel features and investigate their intriguing applications, 3D structures self-assembled by 1D nanostructured units still pose a significant difficulty.

Over the past several years, group II–IV elemental nanomaterials have drawn increasing interest. Mn semiconductors in Group II have undergone thorough study. It was discovered that they exhibit magneto-optical features because of high s and p-d exchange across Mn^2+^ 3d electron states and electron/hole band states [5,6]. Such semiconductors show an anti-ferromagnetic relationship among Mn^2+^ (S = 5/2) spins because of the significant d-p-d interactions. Due to their numerous uses, particularly as a blue/green light emitter, the above semiconductors have recently attracted considerable attention, bolstered by creating amazingly composite structures [7,8]. Manganese sulfide (MnS) compounds have gained more recognition recently for a range of potential applications in optoelectronics, magnetic semiconductors, solar cells, and luminescent sectors. They are essential p-type semiconductors with an enormous band gap energy of 3.7 eV.

Emitting toxic compounds into the air with the development of industries causes serious environmental problems, which can directly affect human lives [9,10]. The global economy and environmental pollution are both heavily influenced by the textile industry, particularly in South African countries [11]. Many scientists and researchers are working on green chemistry and energy to tackle these serious problems. Generally speaking, in water and sediments, some antibodies generate significant problems in the ecological environment, such as tetracycline, which is found in the aquatic environment and affects human and animal health [12,13,14]. Additionally, toxicity and allergy are caused due to antibodies accompanying anti-resistance bacteria in an eco-friendly environment. Despite this, several processes, such as photocatalysis, filtration, adsorption, and biodegradation, are used to eradicate water from the antibodies. These organic contaminations can be effectively detached via a semi-conductor compositing strategy. A number of methods and technologies have already been developed for efficient removal of synthetic dyes from water bodies, including Fenton oxidation, adsorption, reductive degradations using zero-valent metals, biological degradations, and photocatalysis [15,16]. However, semiconductor based photocatalytic degradation is considered as an ecofriendly and cost-effective approach for removal and degradation of organic pollutants, especially synthetic azo-based dyes from wastewater [17]. The higher plasmonic and absorption effects, wider absorption edge, and electron injection rate of the semi-conductor composites make them superior compared to their single components [18,19,20]. Under light irradiation, the semiconductor catalyzes and forms electron-hole (e^−^ and h^+^) pairs on the catalyst surface to start chemical reactions [21]. Therefore, the increased charge carrier separation and light absorption efficiency produce enhanced composite material photocatalytic properties than its pure counterparts [22].

Developing a good photocatalyst is an urgent need in modern society. From this perspective, enormous research has been carried out to search for the best photocatalyst. Various semiconductor materials have been employed as photocatalysts, including TiO_2_, Mn-doped into the ZnS matrix [23], SnO_2_/ZnO [24], and TiO_2_-based ternary composites [25,26,27], graphene oxide (GO), ZnO-CuO composite [28,29,30], graphitic carbon nitride (g-C_3_N_4_)/MnSe [31], etc., that showed good catalytic performance when making composites with other materials. A new class of materials, known as transition metal selenides (TMSs), has gained considerable interest as a photocatalyst under light irradiation owing to their high magnetic, optical, and electrical properties [32,33,34]. Their electron/hole band state and unique structural features are also one of the leading properties that can be ignored for light irradiation [35]. Manganese selenide (MnSe) is one of the attractive TMS that carry some drawbacks when utilized for photoelectrochemical applications. The drawbacks can be effectively overcome when MnSe is combined with other materials, as discussed in the first paragraph of the introduction in detail. GO (graphene oxide) is widely studied as a conductive and good charge carrier separation when combined with any semiconducting material, offering fascinating photocatalytic properties [36,37,38,39,40]. The highly conductive nature of MnSe coupled with electron-hole band structure matching is a perfect match to combine with GO, which signifies the transport and separation of charge carriers.

Nevertheless, the introduction of GO can act as an effect of an electron conductor and an electron accepter that accelerates the interfacial electron transfer process from MnSe, thus strongly hindering the recombination of charge carriers and enhancing the photocatalytic activity. MnS/Ag_2_WO_4_ photocatalyst was recently reported [41] showing a superior photocatalytic efficiency of 92.3% compared with MnS (67.23%) and 58.93% for Ag_2_WO_4_, respectively. MoS_2_-GO nanocomposites were investigated for their photocatalytic activity in methylene blue (MB) breakdown. The degradation of MB by the MoS_2_-GO nanocomposites revealed increased photocatalytic activity, with a maximum rate of 99% within 60 min of solar light irradiation [42].

In this paper, we investigated the photocatalytic performance under organic MB dye for GO-MnSe composite. This study concludes that the 21% GO-MnSe composite presents a better photocatalytic activity under visible light irradiation (80 min) than all other composite materials. These findings demonstrate the promising avenue of TMSs-based carbon composites for energy storage applications.

## 2. Preparation of the Samples

### 2.1. GO Preparation

The GO was synthesized using a modified Hummer’s technique [43], for the typical procedure, a 2.0 g graphite (EG-350-80, Sinopharm Chemical Reagent Co., Ltd., Shanghai, China) powder was added to 100 mL H_2_SO_4_ (95% H_2_SO_4_-pure Sigma Aldrich, St. Louis, MO, USA), and gradually KMnO_4_ (8.0 g) (7122-64-7, 158.04 g/mol KMnO_4_-pure, Sigma Aldrich, St. Louis, MO, USA) was added with continuous stirring. In the above solution, 100 mL of deionized water was added and stirred for another 1 h. The mixture was diluted further by adding 300 mL of deionized water. The remaining KMnO_4_ in suspension was then decreased using 20 mL of 30% H_2_O_2_ (30% H_2_O_2_-pure Sigma Aldrich, St. Louis, MO, USA). This suspension was centrifuged and rinsed with an 800 mL 5% HCl (37% HCl-pure, Sigma Aldrich, St. Louis, MO, USA) aqueous solution and deionized water. The obtained GO was then dried for 24 h at 60 °C.

### 2.2. MnSe Preparation

Manganese selenide was synthesized using the hydrothermal technique [10]. Then, 2.35 g of MnCl_2_(4H_2_O) (99% H_2_SO_4_-pure Sigma Aldrich, St. Louis, MO, USA) were combined in 96 mL deionized water and stirred for 15 min in this synthesis. Separately, a solution of 0.9 g selenium powder in 64 mL ethylenediamine (99% Aladdin Biochemical Co., Ltd. Shanghai, China) was prepared and stirred for 15 min.

After stirring, both solutions were combined and stirred for 1 h. Then, N_2_H_4_·H_2_O (80% H_2_SO_4_-pure Sigma Aldrich, St. Louis, MO, USA) was added drop-by-drop, followed by 15 min of stirring. The brown-colored mixture was placed in a Teflon autoclave for a 15 h hydrothermal reaction at 180 °C. This solution was then centrifuged several times with deionized water and ethanol after drying at 60 °C for 12 h.

### 2.3. GO-MnSe Nanocomposites

By varying the weight percent ratios of GO sheets and MnSe nanostructures, a series of MnSe-GO nanocomposites were created. Sonication was used to disperse 7, 14, 21, and 28% weight ratios of GO (relative to MnSe) and MnSe nanostructures in 50 mL of methanol. This was stirred for approximately thirty minutes before being thoroughly washed with DI water and ethanol. The obtained material was dried overnight in an oven at 60 °C.

### 2.4. Characterization

The structural properties and phase purity of pure MnSe and GO-MnSe nanocomposites were investigated using X-ray diffraction (XRD, produced by Philips) equipped with Cu Kα radiation (λ = 0.15406 nm). The diffraction patterns were found in the 10–80° range. The surface morphologies of all the samples were obtained by scanning electron microscope (SEM, Hitachi S-4700, Tokyo, Japan). The Octane Elite detector is also housed in the equipment for investigating the elemental compositions of the materials. Furthermore, a Fourier transform infrared spectrometer (FTIR, Bruker TENSOR27, Billerica, MA, USA) was used for research relating to functional group analysis. A Shimadzu UV3600 spectrophotometer was used to detect the UV-vis DRS absorption spectra.

### 2.5. Photocatalytic Activity

The organic dye (MB) solution degradation rate examined the photocatalytic activity. A 400 W xenon lamp was used to perform irradiation. The decomposition rate of the organic dye (MB) solution measured the photocatalytic activity. In order to provide for duplicated visible light irradiations, a 400 W Xenon lamp attached with Raman and PL measurement facility DongWoo Optron Co. Ltd., Gwangju, South Korea) was used as a light source. The concentration of MB (50 mg/L) in double distilled water was kept constant for all catalysts. Methylene blue (MB) solution and 0.01 mg of each photocatalyst were used in the reaction process. Prior to the photocatalytic activity, the adsorption/desorption equilibrium between nanoparticles and MB was established by keeping the solution under dark conditions for 30 min with continuous stirring. Once the adsorption/desorption equilibrium was achieved, the Xenon lamp was turned on to initiate the photocatalytic process. The beaker containing nanoparticles and MB was kept at a distance of 15 cm from the lamp to avoid heating of the glass container. The samples from the solution under light irradiation were collected at different time intervals and filtered through 0.45 µm membrane filters for the removal of any suspended particles. A tiny quantity of solution was taken from the reactor after 15 min, and MB degradation was measured using UV–vis spectrophotometer (U-1900 spectrophotometer, Hitachi High-Tech Corporation, Tokyo, Japan). The degradation efficiency was calculated by

(1) $$\%\;\mathrm{Degradation}=\frac{C_{0}-C}{C_{0}}\times 100\%$$

where C_0_ is the beginning dye concentration (mg/L) and C is the final dye concentration (mg/L) after time t (min). As a result, for all observations, a comparison was made between C/C_0_ and photodegradation rate under constant reaction circumstances. For each catalyst, the same procedure was followed.

## 3. Results and Discussion

**Figure 1:**
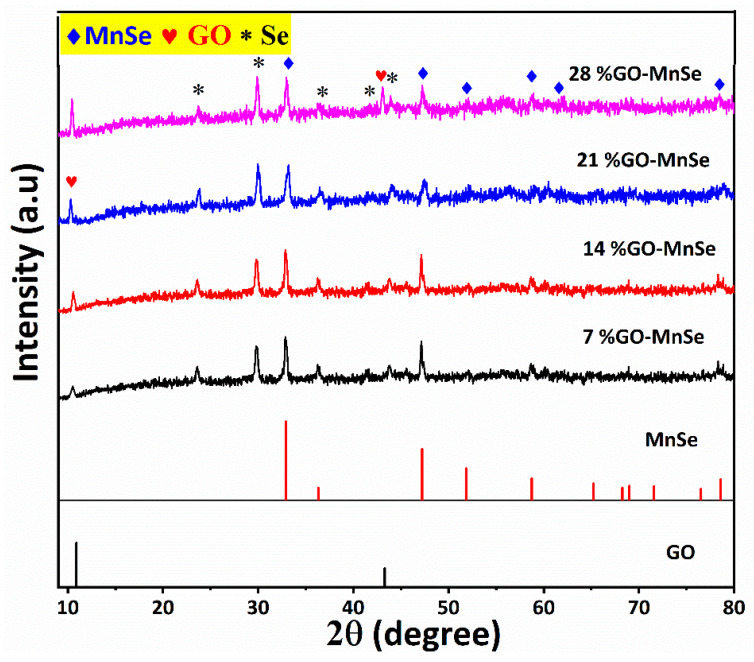
XRD pattern of MnSe, 7% GO-MnSe, 14% GO-MnS, 21% GO-MnSe, and 28% GO-MnSe.

X-ray diffraction (XRD) is the fingerprint of the elements from which we can easily distinguish different nanomaterials. The XRD diffractograms of MnSe and GO-related composites are given in Figure 1. Different colors are used to differentiate their related XRD pattern of individual samples with various compositions. All XRD patterns revealed the corresponding peaks of MnSe at 2 theta values of 32.92°, 36.34°, 47.18°, 51.83°, 54.78°, 68.91°, and 78.44°, which are respectively assigned to (200), (211), (220), (311), (222), (400), (420). The obtained peaks can be well matched with the JCPDS card no. 01-073-1741 [44] and correspond to the cubic phase of MnSe. In addition to the diffraction peaks for MnSe, small peaks are also observed at 23.51°, 29.89°, 41.43°, 43.69°, and 45.59° that can be attributed to the existence of Se impurity and can be indexed to the (100), (101), (110), (102), (111) planes and related to the JCPDS card of Se (00-006-0362). Besides the peaks of MnSe, there is an additional peak observed at 11° with a crystal plane of (002) of GO in all samples. It was perceived that with the increasing GO contents, the peak intensity increases, which leads to the generation of crystalline MnSe structure [44]. No other residue peak was declared in the XRD pattern, implying the high crystallinity and purity of the prepared samples.

**Figure 2:**
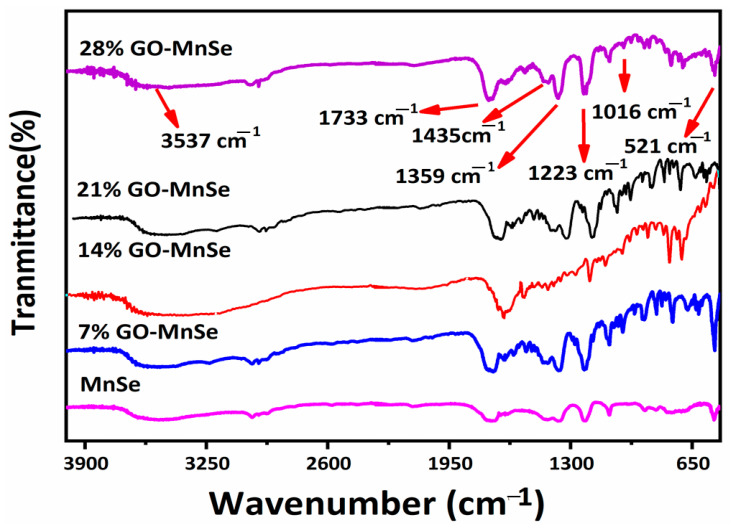
FTIR spectra of MnSe, 7% GO-MnSe, 14% GO-MnS, 21% GO-MnSe, and 28% GO-MnSe.

The Fourier transform infrared spectroscopy (FT-IR) technique can thoroughly study the interaction between matter and infrared radiation. When IR passes through a sample, here in GO-MnSe, depending on its energy, it can trigger a specific molecule’s vibration (vibration). These vibrations include symmetric stretching, ani-symmetric, deformation, rocking, wagging, and twisting. Generally speaking, below 1500 cm^−1^ is considered the fingerprint region, which is unique for each sample and through which we can identify our sample from the database. The opposite side is the functional group region [45]. The FT-IR spectra of the GO-MnSe-based samples are provided in Figure 2. Using FT-IR spectroscopy, the functional groups of the prepared samples were investigated across a range of 600–4000 cm^−1^, the corresponding spectra are displayed in Figure 2. The pure MnSe showed five functional groups in the fingerprint region at different wavenumbers. Mn (II)-Se vibrations may be connected to the band around the wavenumber of 521 cm^−1^ [46]. Similar to the bands at 1016 cm^−1^ and 1223 cm^−1^, the C-O-C stretching vibrations correspond to these frequencies. The bands at 1359, 1435, and 1733 cm^−1^ correspond to the vibration modes of C-O, C=C, and aromatic C=O and C-O-C [47], respectively. The O-H stretching vibration mode is responsible for the broad absorption peak between 3600 and 3800 cm^−1^due to the water molecules stuck to the samples’ surface [48]. Similarly, at several weight ratios of GO (7%, 14%, 21%, and 28%), contents against the MnSe compound were further deeply investigated. We noticed that no clear difference was observed after the introduction of GO into the MnSe matrix.

**Figure 3:**
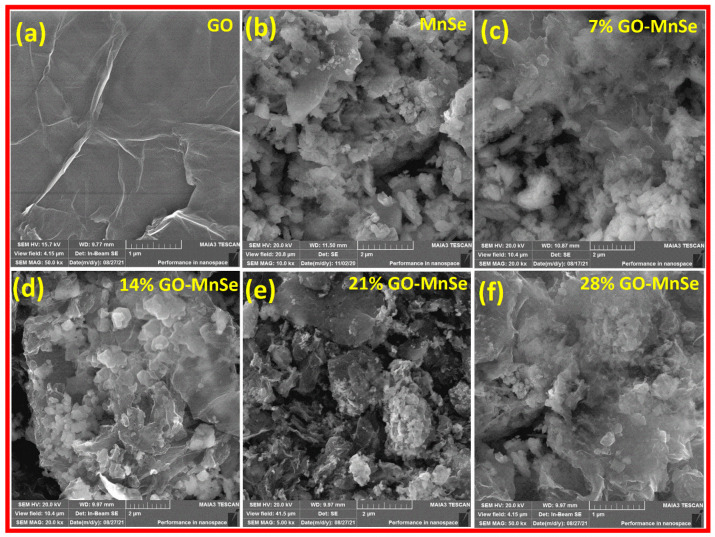
SEM images of (**a**) GO, (**b**) MnSe, (**c**) 7% GO-MnSe, (**d**) 14% GO-MnS, (**e**) 21% GO-MnSe, and (**f**) 28% GO-MnSe.

SEM analysis allows high-resolution imaging to evaluate various materials for surface corrosion, fractures, contaminants, or flaws. The SEM Tamicrographs of all samples are schematically displayed in Figure 3. Pure GO sample (Figure 3a) indicates wrinkled nanosheets composed of aggregation due to the van der Waals force of attraction, which binds GO sheets together [49]. Additionally, the MnSe sample shows a flake-type morphology with different sizes with a dense and closed surface architecture noticed on the MnSe morphology with rough surfaces. According to the XRD investigation, no extra residues were found on the surface, which was also validated from the SEM micrographs, as depicted in Figure 3b, demonstrate the product’s high purity. The effect of different contents of the GO-based MnSe composites, a detailed morphological evaluation, is given in Figure 3c–f. As displayed in Figure 3c, the 7% GO-MnSe composite showed highly aggregated GO sheets on the surface of MnSe. Additionally, the 14 %GO-MnSe composite showed a more uniform distribution of GO on the surface of MnSe than the previous sample (Figure 3d), signaling better photocatalytic performance. In addition, 14% GO-MnSe composite showed the homogenous distribution of GO and eventually covered the MnSe surface (Figure 3e). The GO is seen to be well distributed on the whole surface of the composite material. At the same time, the MnSe arranges in a loose surface architecture, offering highly express roadways and better charge carrier migration. When the GO contents are further increased to 28% (28% GO-MnSe composite), the morphology of the MnSe product seems to be particles hidden with GO sheets.

**Figure 4:**
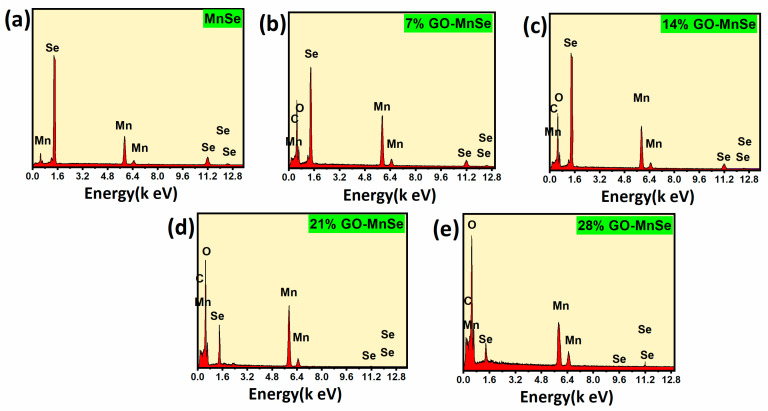
(**a**) EDX spectra of MnSe, (**b**) 7% GO-MnSe, (**c**) 14% GO-MnS, (**d**) 21% GO-MnSe, and (**e**) 28% GO-MnSe.

The compositional analysis of the products was assessed via energy-dispersive X-ray (EDX) analysis. The obtained EDX spectra of all samples are depicted in Figure 4. The EDX spectra of MnSe and GO-MnSe-based composites showed the existence of Mn, Se, and C in their composite without any foreign elements, demonstrating their high purity (see Figure 4a–e), which coincides with SEM and XRD results.

**Figure 5:**
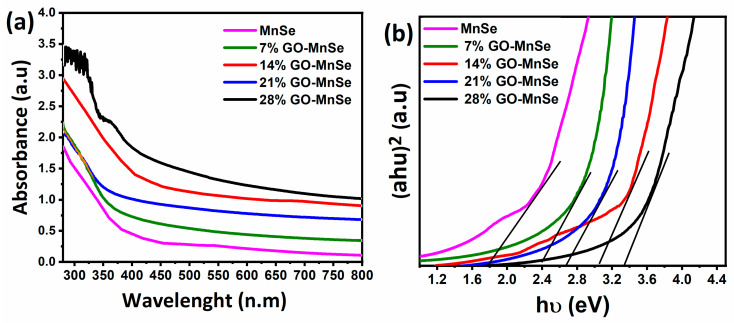
(**a**) DRS spectra (**b**) bandgap energy of MnSe, 7% GO-MnSe, 14% GO-MnS, 21% GO-MnSe, and 28% GO-MnSe.

The optical properties of the samples were studied further since a semiconductor with excellent photocatalytic efficiency should have a broad spectrum of light absorption. Several methods, including UV-vis absorption spectroscopy and optical bandgap, were used to investigate the optoelectronic properties of the 7% GO-MnSe, 14% GO-MnS, 21% GO-MnSe, and 28% GO-MnSe based on MnSe, GO, and MnSe. As shown in Figure 5a, UV-Vis spectroscopy is an essential method for researching the optoelectronic properties of semiconducting materials such as MnSe and 7% GO-MnSe, 14% GO-MnS, 21% GO-MnSe, and 28% GO-MnSe. MnSe has an absorption peak at 368 nm compared to other contents of GO with MnSe (351 nm), with a prominent absorption edge, exhibiting the characteristic bandgap absorption of nanomaterials in the UV-vis range caused by electron transfer from VB to CB. More specifically, the absorption of 7% GO-MnSe, 14% GO-MnS, 21% GO-MnSe, and 28% GO-MnSe absorption zones shows that the combination of MnSe is more important in the combination than in the pure samples. The bandgap is computed for MnSe (1.7 eV), 7% GO-MnSe (2.42 eV), 14% GO-MnSe (2.6 eV), 21% GO-MnSe (3.02 eV), and 28% GO-MnSe (3.24 eV) as shown in the Tauc plots (Figure 5b), which is generally comparable to the relevant literature findings [32,50,51]. The resulting bandgap energy is plotted versus MnSe concentrations, indicating that as GO molar content increases, so does the bandgap energy.

**Figure 6:**
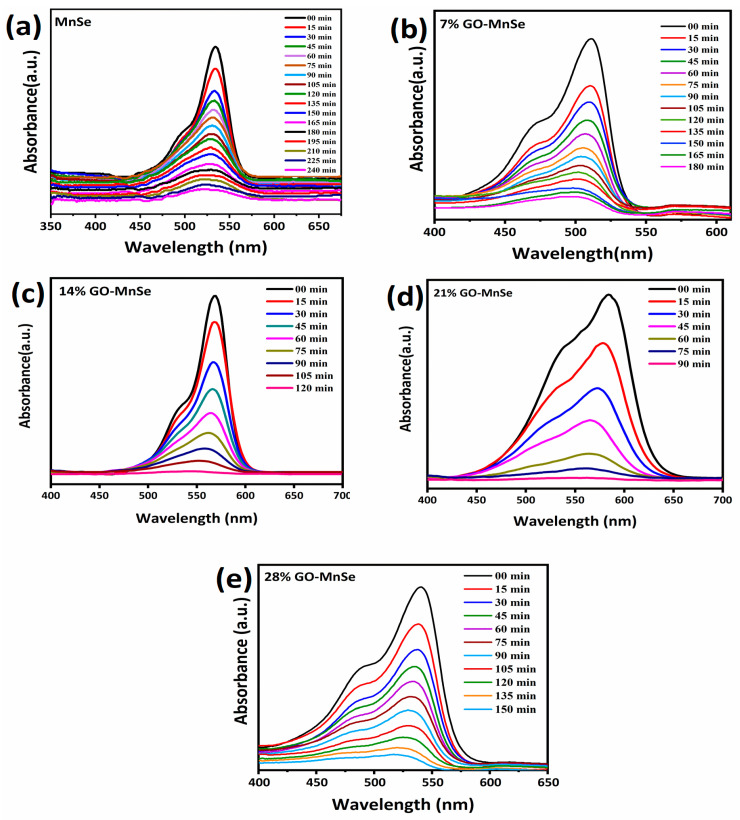
UV-vis spectra of (**a**) MnSe, (**b**) 7% GO-MnSe, (**c**) 14% GO-MnSe, (**d**) 21% GO-MnSe, (**e**) 28% GO-MnSe.

**Figure 7:**
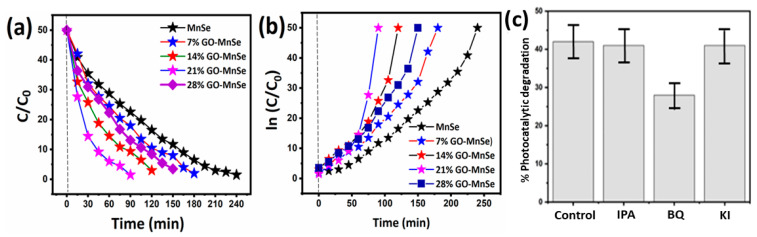
(**a**,**b**) Concentration changes of MB as a function of irradiation time during the degradation process by nanocomposites MnSe, 7% GO-MnSe, 14% GO-MnS, 21% GO-MnSe, and 28% GO-MnSe plots, and (**c**) photodegradation of MB over sepiolite in the presence of different scavengers, i.e., isopropanol (IPA), benzoquinone (BQ), and potassium iodide (KI).

In the remediation of the environment, the effective degradation of organic dye has long been a challenge. Therefore, to assess the photocatalytic activity of the materials under visible light irradiation, MB was utilized as the target organic pollutant. The comparative UV-vis absorption spectroscopy results of the wt.% GO-MnSe composite are shown in Figure 6. Compared with the pure MnSe sample, their composite revealed enhanced absorption at the wavelength of 550 nm. The 21% GO-MnSe composite showed maximum absorption among different composites, indicating good photocatalytic performance. To further understand the performance of each composite, the detailed UV-absorption spectroscopy results for individual samples are provided in Figure 6. Pure MnSe takes 240 min to degrade the MB dye to clean the wastewater. Notably, after adding GO, the MB dye degradation time reduced, meaning that the GO introduction accelerated the catalyst process. Similarly, the 7% GO-MnSe and 14% GO-MnSe composites achieved a total time of 180 min and 120 min (Figure 6b,c), which is better the pure MnSe, but it cannot be considered a good catalyst. As progressively, the 21% GO-MnSe composite displayed the fastest test degradation of organic MB dye at 90 min (Figure 6d), indicating the good photocatalytic performance of the composite material. When further the GO contents (28% GO-MnSe composite) were further increased, the degradation time of the MB dye further upsurged to 150 min (Figure 6e). This means that the optimal ratio in this work is 21% GO, making the photoelectrochemical process faster than all other samples. We believe that our work, which is shown in Figure 6, reveals impressive photocatalytic degradation of MB and presents a promising candidate for environmental remediation. The high adsorption % of the 21% GO-MnSe is also attributed to their shape, gaps/cracks, and open network structure. The variously reported photocatalysts for dye degradation are shown in Table 1. It was observed that the 21% GO-MnSe composite is highly effective, and its degradation rate was superior to or similar to those described in the literature (Table 1). For determining the role of responsible reactive species for the degradation of RhB, different radical scavengers such as isopropanol (IPA), benzoquinone (BQ), and potassium iodide (KI) were used for scavenging h^+^, ^•^O_2_^−^, and ^•^OH, respectively, as depicted in (Figure 7c) [52,53]. A significant decrease in degradation efficiency was observed due to entrapping of ^•^O_2_^−^ by BQ; however, in the case of KI and IPA, a negligible decrease in degradation efficiency was observed. This clearly indicates that ^•^O_2_^−^ is the main oxidant specie that plays a vital role in the degradation of RhB as compared to h^+^ and ^•^OH.

To further investigate the enhanced photocatalytic activities of the samples, the standard model is shown in Figure 7 against time vs. C/C_0_ degradation results. The rate constant for each sample is schematically given in Figure 7a,b. The resultant graph in Figure 7a,b confirms the sample’s enhanced catalytic ability with 21% GO mixed with MnSe. This further indicates that, on the surface of 21% GO-MnSe, the free radicals interact with a couple of redox reactions, producing an eco-friendly compound after the oxidation of MB dye. The economic ease of the synthesis protocol of the GO-MnSe sample leads to the beneficial removal of other organic compounds from wastewater and water. The Langmuir–Hinshelwood (L-H) kinetic model, which is given by kinetic Equation (2) for lower concentrations, is often utilized for the photocatalytic degradation of different organic dyes.

(2) $$\ln=\left(\frac{C}{C_{0}}\right)=\mathrm{kt}$$

where k is the rate constant, C_0_ is the initial concentration, C is the final concentration following irradiation time t, and the plot of C/C_0_ against irradiation time is the accepted technique for displaying deterioration findings. Figure 7 displays the C/C_0_ plot against irradiation time along with the reaction rate constants for each sample and following irradiation time intervals derived using the L-H model. In our scenario, based on the above studies for Figure 7, the pseudo first order reaction rate constant is taken into consideration due to the decreased concentration of MB.

**Scheme 1:**
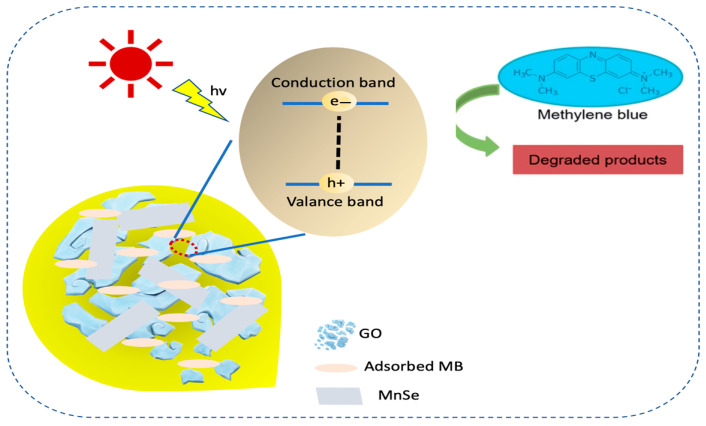
The schematic representation of the degradation and absorption mechanism of the MB under visible light.

In a photocatalytic reaction, when a photon with energy equal to or greater than the energy of the band gap of semiconductor strikes on the surface of the semiconductor, it causes photoexcitation of electrons. The photoexcited electron (e^−^) then jumps from valence band (VB) to a higher-level conduction band (CB), generating a positive hole (h^+^) in VB. Thus, the e^−^/h^+^ pairs created at the surface of photocatalyst during the process are responsible for photocatalytic degradation of pollutants [59]. A suitable band gap, large surface area, low cost, high efficiency, and recyclability are the characteristics of an ideal photocatalyst [9]. To better understand the process of MnSe-GO composite photocatalytic breakdown and absorption into MB under UV light, a graphic depiction was presented in Scheme 1. The MnSe-GO composite with efficient catalytic and adsorption efficiency can be attributed to its special structure, which has the accompanying advantageous characteristics:

- The covalent MB molecules might connect to huge aromatic domains on GO layers via p-p stacking, favoring a significant increase in reactivity [60] and efficiently improving light absorption in the visible region.
- Our excellent photocatalytic and repeatable performance might be attributed to the tight coupling between MnSe and GO, which enhances interface charge transfer (in GO as an acceptor) and prevents electron-hole coupling [61].

## 4. Conclusions

This work utilizes a facile synthesis of different wt.% GO-MnSe based composites and their photoelectrochemical performance was tested against MB dye. Adequate and facile preparation of the samples was confirmed through XRD, SEM, and EDX investigations with high purity and good structural integrity. The morphological study showed the introduction of GO into the MnSe matrix, which greatly enhances the conductive support during light irradiation. Additionally, FT-IR further depicted the presence of host material with the presence of functional group appearance in the combined spectra. The bandgap absorption analysis derived from optical properties demonstrated that the bandgap energy reduces substantially, resulting in enhanced photocatalytic performance. The best sample of 21 %GO-MnSe composite illustrates the enhanced photocatalytic performance compared with others, proving the promising usage of selenide-based carbon composite for future energy storage devices.

## Figures and Tables

**Table 1:** Comparison of our work with other previously reported literature.

Material	Light Source	Irradiation Time (min)	MB Reduction (%)	Ref.
ZnO/rGO	Visible light	120	85	[54]
TiO_2_/GO	UV light	150	88	[55]
CQDs-TiO_2_-x-rGO	Visible light	120	91	[56]
Ag-TiO_2_/rGO	Visible light	100	93	[57]
CuSe	Visible light	90	76	[58]
MnSe	Visible light	90	--	This work
7 %MnSe/GO	Visible light	90	--	This work
14 %MnSe/GO	Visible light	90	--	This work
21 %MnSe/GO	Visible light	90	--	This work
28 %MnSe/GO	Visible light	90	--	This work

## Data Availability

Data is contained within the article.
